# Extracorporeal Life Support Enhances the Forward Pressure Wave to Cause a Mismatch between Cardiac Oxygen Demand and Supply

**DOI:** 10.1038/s41598-019-50428-1

**Published:** 2019-09-25

**Authors:** Chih-Hsien Wang, Ru-Wen Chang, En- Ting Wu, Yi-Jing Hsiao, Ming-Shiou Wu, Hsi-Yu Yu, Yih-Sharng Chen, Liang-Chuan Lai, Sung-Liang Yu

**Affiliations:** 1 Cardiovascular Surgery, Department of Surgery, National Taiwan University Hospital, Taipei, Taiwan; 2 Department of Pediatrics, College of Medicine, National Taiwan University Hospital, Taipei, Taiwan; 3 Department of Clinical and Laboratory Sciences and Medical Biotechnology, National Taiwan University College of Medicine, Taipei, Taiwan; 4 Department of Internal Medicine, National Taiwan University Hospital, Taipei, Taiwan; 5 Department of Physiology, College of Medicine, National Taiwan University, Taipei, Taiwan

**Keywords:** Cardiovascular biology, Applied mathematics

## Abstract

Extracorporeal life support (ECLS) is a world-famous life-saving method. Until now, changes in arterial wave properties due to ECLS have remained unexamined. In this study, we determined the effects of ECLS on arterial wave properties and ventricular/arterial coupling in male Wistar rats with the measured aortic pressure alone. Ascending aortic pressure signals were measured before ECLS and at 30, 60, and 90 min after weaned off. The aortic pressure signal then calculated by fourth-order derivative to obtain an assumed triangular flow wave. The ratio of mean systolic pressure to mean diastolic pressure (*P*_*ms*_/*P*_*md*_), a parameter for evaluating the matching condition between myocardial oxygen demand and supply, was significantly higher after ECLS. The magnitude of forward pressure (|*P*_*f*_|) augmented by ECLS prevailed over the backward pressure (|*P*_*b*_|), leading to a decline in wave reflection factor. *P*_*ms*_/*P*_*md*_ was positively linearly correlated with |*P*_*f*_| (*P*_*ms*_/*P*_*md*_ = 0.9177 + 0.0078 × |*P*_*f*_|, *r* = 0.8677; *P* < 0.0001). These findings suggest that |*P*_*f*_| was a predominant factor responsible for the mismatch between the myocardial oxygen demand and supply in rats after ECLS phase of experiment.

## Introduction

Extracorporeal life support (ECLS) has become a life-saving method for adults^1,2^ and children^3,4^ worldwide since its first successful use in 1971^5^. The implementation usually involves femoral cannulation due to its simplicity, efficiency, and ease of use outside the operating room^6^. Although the success rates of ECLS have improved, it still causes some severe side effects. Reports from clinical investigations have indicated that management with ECLS might induce significant complications, such as acute kidney injury^7^, bleeding^8^, and inflammatory insults^8^. Moreover, femoral cannulation causes massive retrograde aortic ECLS flow^6,9^, causing a watershed of the cardiac and ECLS flows in the aortic arch during ECLS^9^. Whether the massive retrograde aortic ECLS flow might exert deteriorative effects on arterial wave properties and matching condition of oxygen demand and supply in myocardium has not been investigated until now.

The accurate measurement of the pulsatile nature of the arterial system, including arterial wave transit time (*τ*_*w*_) and the forward (*P*_*f*_) and backward (*P*_*b*_) pressure waves, requires simultaneous recording of aortic pressure and flow signals^10–12^. However, it is difficult to steadily measure the ascending aortic flow signal in rats and humans before, during, and after ECLS. In 2006, Westerhof *et al*.^13^ described and validated that the aortic flow can be approximated by a triangle (*Q*^tri^), which is constructed from the measured aortic pressure waveform. Based on aortic pressure and *Q*^tri^, *P*_*f*_ and *P*_*b*_, components of aortic pressure wave, were successfully obtained by the wave separation method. Moreover, Chang *et al*.^14^ discovered that the aortic impulse response is effective for estimating arterial *τ*_*w*_ by a single pressure pulse with its corresponding *Q*^tri^. The novelty of the concept proposed by Westerhof *et al*. is that the constructed *Q*^tri^, approximately to its paired flow signal, is based on the measured aortic pressure wave, and the calibration is not required in the analysis^13^.

Ventricular/arterial coupling concerns two matters, action of the heart as a pump connected to a hydraulic load and perfusion of the heart as an organ. In 1972, Buckberg *et al*.^15^ showed that an index based on LV and aortic pressures could predict subendocardial ischemia: the area between the diastolic aortic and LV pressures (DPTI) represented the oxygen supply to the myocardium, and the area under the systolic LV pressure curve (SPTI) represented the oxygen demand by the myocardium. As pressure in the ventricle during systole is approximated by aortic pressure during systole, for practical purpose, O’Rourke^10^ and O’Rourke *et al*.^16^ suggested that ventricular function as pumping action could be described using mean pressure generated in the ascending aorta during systole. Thus, the ratio of mean systolic aortic pressure (*P*_*ms*_) to the mean diastolic aortic pressure (*P*_*md*_) could be an indicator for evaluating the matching condition between myocardial oxygen demand and supply.

In this study, we used the measured aortic pressure and an assumed *Q*^tri^ to determine the effects of ECLS on arterial wave properties and ventricular/arterial coupling in male Wistar rats. The construction of *Q*^tri^ was derived from the fourth-order derivative of the measured aortic pressure wave^13,14,17^. The aortic input impedance (*Z*_*i*_) was calculated from the ratio of the ascending aortic pressure harmonics to the corresponding *Q*^tri^ harmonics. The arterial *τ*_*w*_ was determined by the aortic impulse response, which is the time-domain equivalent of its *Z*_*i*_ in the frequency-domain. The *P*_*f*_ and *P*_*b*_ components of the pressure pulse were separated using the wave decomposition technique. Thus, the arterial *τ*_*w*_, and magnitudes of the forward (|*P*_*f*_|) and backward (|*P*_*b*_|) pressure waves were derived from the only measured aortic pressure to delineate the changes caused by ECLS in the pulsatile nature of the LV afterload.

## Results

### Hemodynamic parameters compared between baseline and post-ECLS 30, 60, 90 min

The mean body weight of the rats was 500 ± 35 g. Table 1 shows the basal heart rate (HR), cardiac cycle length (CL), LV ejection time (LVET), and aortic pressure profiles before and after ECLS treatment. After weaning from ECLS, the rats had increased HR, decreased CL, and shortened LVET. By contrast, no significant difference was observed in these parameters between post-ECLS 30, 60, and 90 min. The systolic (*P*_*s*_), diastolic (*P*_*d*_), and mean (*P*_*m*_) aortic blood pressures did not change significantly in these rats after ECLS. By contrast, the *PP* values were markedly higher at post-ECLS 30, 60, and 90 min than at baseline.

**Table 1:** Basic hemodynamic data measured in rats at baseline and at 30, 60, and 90 min post-ECLS.

	HR	CL	LVET	*P*_*s*_	*P*_*d*_	*P*_*m*_	*PP*
**Time point**							
Baseline	366.5 ± 27.1	163.7 ± 12.5	60.6 ± 5.2	113.0 ± 20.4	80.8 ± 20.6	98.3 ± 19.7	31.7 ± 7.2
Post-ECLS 30 min	390.8 ± 22.2^*^	153.5 ± 8.8^*^	56.4 ± 6.1^*^	123.6 ± 18.2	79.4 ± 30.5	100.7 ± 33.1	44.4 ± 12.5^*^
Post-ECLS 60 min	398.4 ± 33.8^*^	150.6 ± 12.7^*^	53.7 ± 5.8^*^	122.8 ± 18.9	79.9 ± 23.6	100.9 ± 24.1	43.1 ± 15.0^*^
Post-ECLS 90 min	394.9 ± 49.3^*^	151.9 ± 18.5^*^	53.3 ± 7.0^*^	122.9 ± 22.0	81.2 ± 31.0	104.1 ± 31.0	42.4 ± 14.6^* † #^

**Figure 1:**
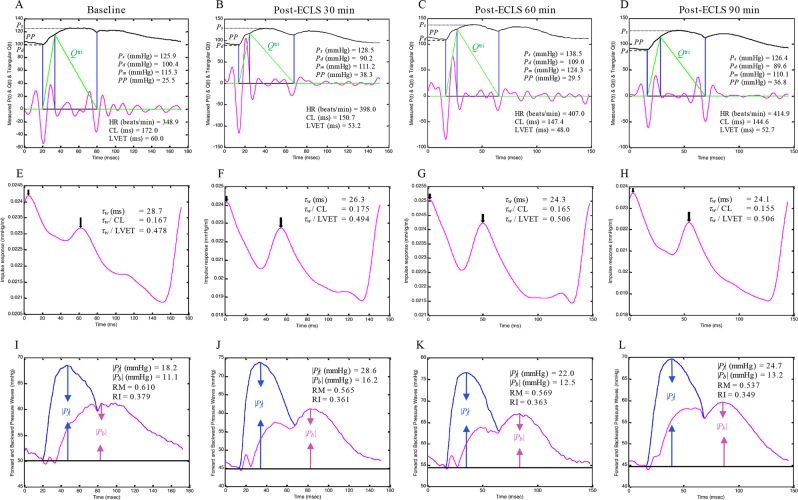
Construction of an uncalibrated *Q*^tri^ derived from the measured pressure waveform in a rat at baseline (**A**) and at 30 (**B**), 60 (**C**), and 90 (**D**) min post-ECLS. In (**E–H**), the aortic impulse response function curve derived from the measured aortic pressure and its assumed *Q*^tri^. In (**I–L**), the amplitudes (peak – trough) of the *P*_*f*_ and *P*_*b*_ are described by |*P*_*f*_| and |*P*_*b*_|, respectively. CL = cardiac cycle length; HR = basal heart rate; LVET = left ventricular ejection time; *P*_*b*_ = backward pressure wave; *P*_*d*_ = diastolic pressure; *P*_*f*_ = forward pressure wave; *P*_*m*_ = mean aortic pressure; *P*_*s*_ = systolic pressure; *PP* = pulse pressure; *Q*^tri^ = triangular flow; RM = wave reflection magnitude, given by |*P*_*b*_|/|*P*_*f*_|; RI = wave reflection index, given by |*P*_*b*_|/(|*P*_*f*_| + |*P*_*b*_|); *τ*_w_ = wave transit time.

Variables are expressed as median ± interquartile range (IQR). HR = heart rate (beats min^−1^); CL = cardiac cycle length (ms, which means the duration of single beat); LVET = left ventricular ejection time (ms, which was derived from the duration of the first two vertical lines in Fig. 1A–D); *P*_*s*_ = systolic pressure (mmHg); *P*_*d*_ = diastolic pressure (mmHg); *P*_*m*_ = mean pressure (mmHg); *PP* = pulse pressure (mmHg).

*P < 0.05 compared with baseline. ^†^*P* < 0.05 compared with post-ECLS 30 min. ^#^*P* < 0.05 compared with post-ECLS 60 min.

Table 2 shows the mean systolic (*P*_*ms*_), and mean diastolic (*P*_*md*_) aortic blood pressures and the fold changes compared with its baseline. They did not change significantly after ECLS. However, the *P*_*ms*_/*P*_*md*_ ratio significantly increased following ECLS and markedly decreased at post-ECLS 60 and 90 min compared with 30 min.

**Table 2:** Effects of ECLS on the mean systolic and the mean diastolic pressure in rats at baseline and at 30, 60, and 90 min post-ECLS.

	*P*_*ms*_	Fold change	*P*_*md*_	Fold change	*P*_*ms*_/*P*_*md*_
**Time point**					
Baseline	106.0 ± 19.7	1.000 ± 0.000	93.5 ± 20.8	1.000 ± 0.000	1.114 ± 0.058
Post-ECLS 30 min	110.2 ± 29.3	1.063 ± 0.219	91.3 ± 34.0	0.949 ± 0.383	1.169 ± 0.118^*^
Post-ECLS 60 min	110.2 ± 21.0	1.064 ± 0.227	95.9 ± 25.1	1.038 ± 0.343	1.157 ± 0.104^* †^
Post-ECLS 90 min	113.7 ± 26.5	1.105 ± 0.205	95.6 ± 32.1	1.003 ± 0.400	1.155 ± 0.101^* †^

Variables are expressed as median ± interquartile range (IQR). *P*_*ms*_ = mean systolic pressure (mmHg); *P*_*md*_ = mean diastolic pressure (mmHg).

*P < 0.05 compared with baseline. ^†^*P* < 0.05 compared with post-ECLS 30 min.

### Pulse wave reflection changed by ECLS

Table 3 shows the pulse wave reflection obtained from the aortic pulsatile pressure signal in rats before and after ECLS. After ECLS, the rats had significantly higher |*P*_*f*_| and |*P*_*b*_| than before ECLS. The fold changes of |*P*_*f*_| and |*P*_*b*_| also significantly increased after ECLS, with the fold change level of |*P*_*f*_| being higher than that of |*P*_*b*_|. Although there was a trend toward decreasing arterial *τ*_*w*_ in rats following ECLS, the *τ*_*w*_/CL ratio remained unaltered.

**Table 3:** Effects of ECLS on the pulse wave reflection obtained from aortic pressure waveform in rats at baseline and at 30, 60, and 90 min post-ECLS.

	|*P*_*f*_|	Fold change	|*P*_*b*_|	Fold change	*τ*_*w*_	*τ*_*w*_/CL
**Time point**						
Baseline	22.5 ± 6.7	1.000 ± 0.000	12.4 ± 2.1	1.000 ± 0.000	30.1 ± 3.0	0.180 ± 0.016
Post-ECLS 30 min	32.5 ± 14.5^*^	1.594 ± 0.505^*^	16.0 ± 2.2^*^	1.326 ± 0.248^*^	26.7 ± 2.6	0.178 ± 0.020
Post-ECLS 60 min	33.8 ± 15.7^*^	1.611 ± 0.395^*^	15.7 ± 2.2^*^	1.232 ± 0.210^*^	26.5 ± 3.0	0.182 ± 0.022
Post-ECLS 90 min	31.7 ± 14.0^*^	1.467 ± 0.497^*^	14.9 ± 2.7^*^	1.208 ± 0.231^*^	26.6 ± 3.0	0.180 ± 0.022

Variables are expressed as median  ±  interquartile range (IQR). CL = cardiac cycle length (ms); |*P*_*f*_| = magnitude of forward pressure wave (mmHg); |*P*_*b*_| = magnitude of reflected pressure wave (mmHg); *τ*_*w*_ = arterial wave transit time (ms).

*P < 0.05 compared with baseline.

### The constructed *Q*^tri^ derived from the measure pressure wave in one rat

Figure 1 shows the construction of an uncalibrated *Q*^tri^ obtained from the measured pressure wave in a rat at baseline (A) and at 30 (B), 60 (C), and 90 (D) min post-ECLS. The aortic pressure wave was measured from the ascending aorta under anesthesia and in the closed-chest condition. Aortic impulse response function curve (Fig. 1E–H) obtained from the measured aortic pressure with its paired *Q*^tri^. In I-L, the amplitudes (peak – trough) of the *P*_*f*_ and *P*_*b*_ are represented by |*P*_*f*_| and |*P*_*b*_|, respectively.

### *P*_*f*_ dominated the increase in *P*_*ms*_/*P*_*md*_ ratio, which can also be easily detected by *PP*, after ECLS

**Figure 2:**
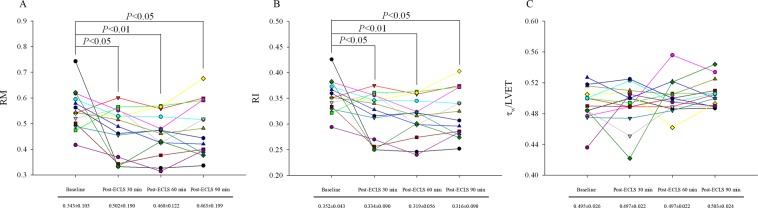
Effects of ECLS on the changes of RM (**A**), RI (**B**), and *τ*_w_/LVET (**C**). Each line represents a different rat in this study. RM and RI show downward trends, which decreased markedly after ECLS treatment. The *τ*_w_/LVET ratio tended to slightly increase after ECLS treatment. RI = wave reflection index; RM = wave reflection magnitude; *τ*_w_/LVET = the ratio of wave transit time to left ventricular ejection period.

Figure 2 illustrates the performance of ECLS on the arterial wave properties including RM, RI, and *τ*_*w*_/LVET ratio. The RM (Fig. 2A) and RI (Fig. 2B) values at baseline were significantly higher than at post-ECLS 30, 60, and 90 min. By contrast, the *τ*_*w*_/LVET ratio did not significantly change before and after ECLS (Fig. 2C).

**Figure 3:**
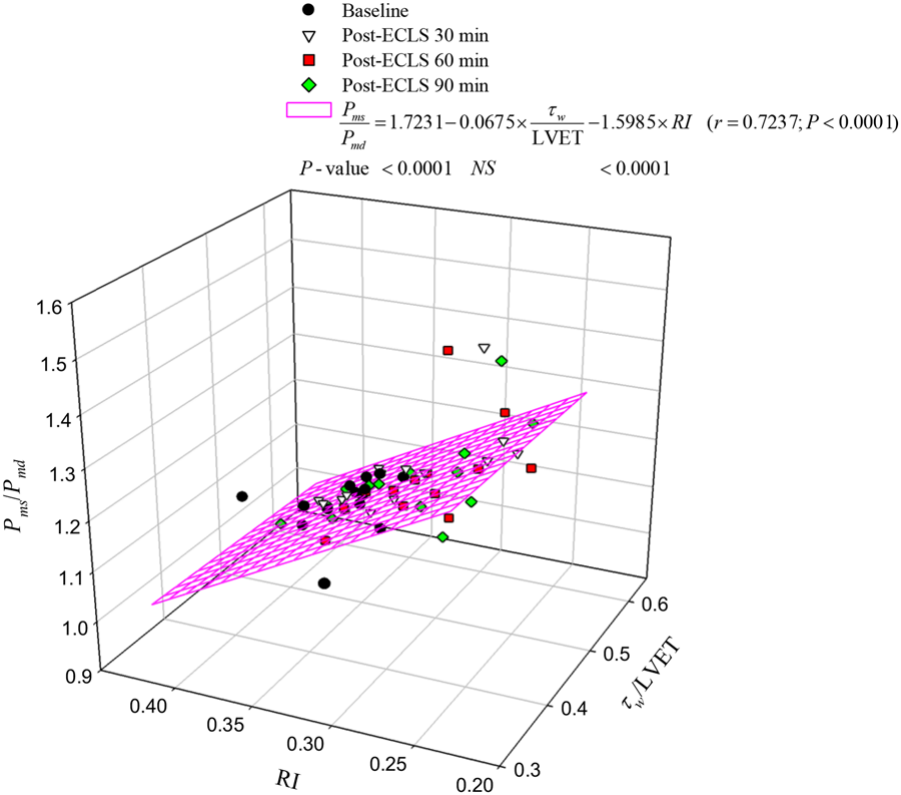
Implication of *τ*_w_/LVET and RI in *P*_*ms*_/*P*_*md*_ ratio in rats before and after ECLS. As shown by multiple linear regression analysis, only the correlation between *P*_*ms*_/*P*_*md*_ and RI reached significance, suggesting that the *P*_*ms*_/*P*_*md*_ impaired by ECLS could be affected by the magnitude of RI rather than the wave transmission time (*τ*_w_/LVET). *P*_*md*_ = mean diastolic pressure; *P*_*ms*_ = mean systolic pressure; RI = wave reflection index; *τ*_w_/LVET = the ratio of wave transit time to left ventricular ejection period.

**Figure 4:**
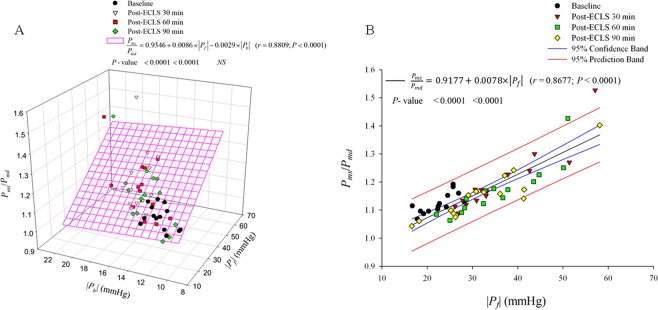
|*P*_*f*_| but not |*P*_*b*_| strongly correlated with *P*_*ms*_/*P*_*md*_ in rats before and after ECLS. As shown by multiple linear regression analysis, the correlation between *P*_*ms*_/*P*_*md*_ and |*P*_*f*_| and |*P*_*b*_| reached significance. (**A**) However, only |*P*_*f*_| was significantly positively related to *P*_*ms*_/*P*_*md*_ before and after ECLS (**B**), indicating that the *P*_*ms*_/*P*_*md*_ impaired by ECLS might be influenced by |*P*_*f*_| rather than |*P*_*b*_|. *P*_*b*_ = backward pressure wave; *P*_*f*_ = forward pressure wave; *P*_*md*_ = mean diastolic pressure; *P*_*ms*_ = mean systolic pressure.

Figure 3 shows the prediction of *P*_*ms*_/*P*_*md*_ ratio from the *τ*_*w*_/LVET ratio and RI before and after ECLS. Taking *P*_*ms*_/*P*_*md*_ as the dependent variable and arterial *τ*_*w*_/LVET and RI as independent variables, multiple linear regression was employed to fit the data. Only the correlation between *P*_*ms*_/*P*_*md*_ and RI reached significance. The influences of |*P*_*f*_| and |*P*_*b*_| on *P*_*ms*_/*P*_*md*_ ratio are depicted in Fig. 4A. The correlation between *P*_*ms*_/*P*_*md*_ and |*P*_*f*_| (but not |*P*_*b*_|) achieved significance. Figure 4B shows the positive linear relationship between the *P*_*ms*_/*P*_*md*_ and |*P*_*f*_| in rats before and after ECLS (*P*_*ms*_/*P*_*md*_ = 0.9177 + 0.0078 × |*P*_*f*_| (*r* = 0.8677; *P* < 0.0001).

**Figure 5:**
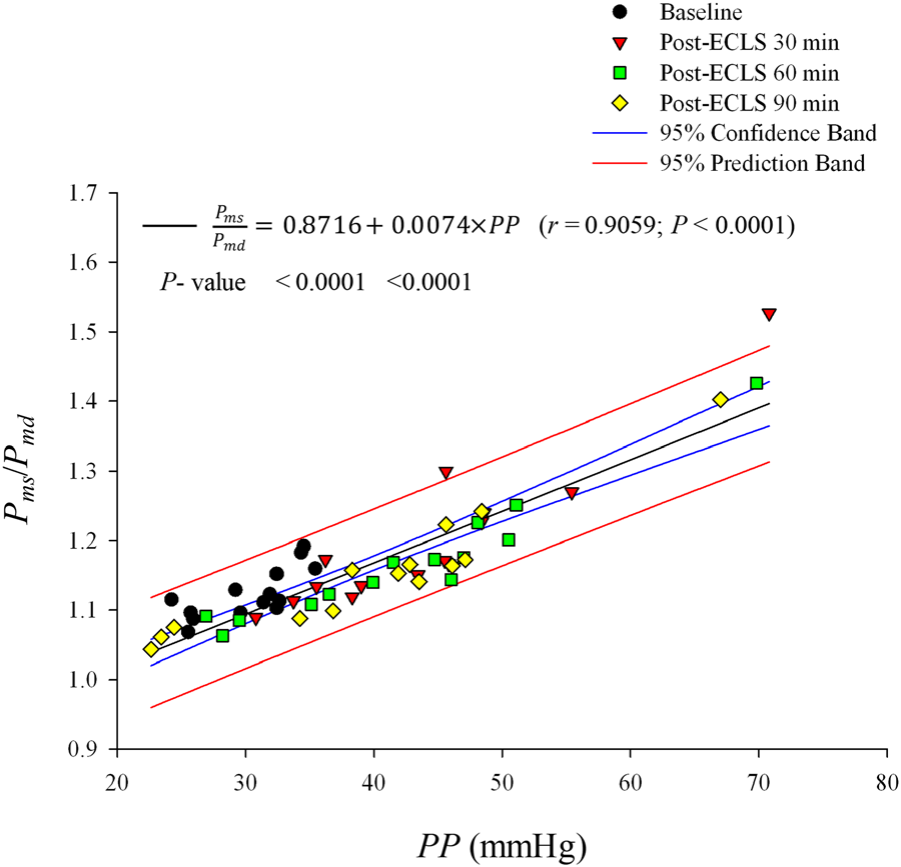
Potential role of *PP* in reflecting *P*_*ms*_/*P*_*md*_ in rats before and after ECLS. *P*_*ms*_/*P*_*md*_ was significantly positively related to *PP* by ECLS. *P*_*md*_ = mean diastolic pressure; *P*_*ms*_ = mean systolic pressure; *PP* = pulse pressure.

Figure 5 displays the relationship of *P*_*ms*_/*P*_*md*_ ratio and *PP* in rats before and after ECLS. *PP* had a significantly positive regression with *P*_*ms*_/*P*_*md*_: *P*_*ms*_/*P*_*md*_ = 0.8716 + 0.0074 × *PP* (*r* = 0.9059; *P* < 0.0001).

## Discussion

Although ECLS can be used as a bridge to recovery for patients with severe heart failure^18^ or heart transplantation^19^, this technology is associated with several side effects, such as blood cell consumption and activation of the cytokine network and the complement cascade systems^20,21^. Theoretically, retrograde aortic ECLS flow can increase cardiac afterload, thus leading to LV distension and endangering ventricular recovery^9^. However, little attention has been given to the pulsatile hemodynamic response to ECLS, which disrupts the ventricular/arterial coupling. To the best of our knowledge, this is the first study suggesting that ECLS can enhance |*P*_*f*_|, the predominant factor responsible for the mismatch between the myocardial oxygen demand and supply.

In this study, the rats exhibited increased HR and shortened LVET after ECLS. We had previously observed high levels of inflammatory cytokines in rats at 120 min after reperfusion^22^, demonstrating persistent inflammatory insults, even after the rats had been weaned from ECLS. These inflammatory responses can be induced by the adrenergic pro-inflammatory pathway^23^, which might cause accelerated heartbeat after ECLS^22^. However, ECLS exerted no significant alterations in aortic pressure profiles such as *P*_*s*_, *P*_*d*_, *P*_*m*_, *P*_*ms*_, and *P*_*md*_ except *PP* and *P*_*ms*_/*P*_*md*_.

The arterial *τ*_*w*_ can be determined by the pulse wave velocity (*PWV*) and traveling distance of pressure waves to the reflecting site^24^. In this study, we calculated the arterial *τ*_*w*_ using the aortic impulse response, which presented the two discrete reflection peaks respectively linked to the effective reflection sites (Fig. 1E–H)^25^. Since CL was a factor influencing the calculation of arterial *τ*_*w*_, the *τ*_*w*_/CL ratio was derived as an index for the description of aortic stiffness. Neither arterial *τ*_*w*_ nor *τ*_*w*_/CL changed significantly after the rats had been weaned from ECLS, indicating that ECLS did not alter vasculature distensibility. Moreover, the return time of |*P*_*b*_| occurred during diastole in rats before and after ECLS (Fig. 1I–L). Both |*P*_*f*_| and |*P*_*b*_| were increased by ECLS (Table 3). However, the predominant role in increasing |*P*_*f*_| rather than |*P*_*b*_| contributed to a decline in RM (Fig. 2A) and RI (Fig. 2B). Although controversial, the prevalent view considers that the |*P*_*f*_*|* value primarily reveals the interaction between peak aortic flow and proximal aortic diameter/stiffness while minimal/delayed reflections occur^26–28^. Thus, the increased |*P*_*f*_| in the absence of changes in both *τ*_*w*_ and *τ*_*w*_/CL indicated that ECLS may cause a functional mismatch between peak flow and physical properties of the proximal aortas.

The LV-arterial system interaction is an essential determinant of cardiovascular function and plays a crucial role in various cardiovascular disease states. Scholars have expressed great interest in characterizing the LV-arterial coupling by only measuring the ascending aortic pressure. Since coronary flow to the LV wall can occur only during diastole, the coronary perfusion pressure is the pressure gradient between the aorta and the LV during diastole, when the myocardium receives oxygen. However, the pressure generated by the left ventricle during systole is the pressure that opposes ejection of blood from the ventricle, and that determines myocardial oxygen requirements^29^. As mentioned previously, O’Rourke *et al*.^16^ suggested that the ascending aortic pressure wave comprises two components: *P*_*ms*_, describing the LV performance as a pump, and *P*_*md*_, relating to LV perfusion. Depending on this, the “ideal” ventricular/arterial coupling for adequate organ flow needs to meet as low a *P*_*ms*_ and as high a *P*_*md*_ as possible. Low *P*_*ms*_ allows adequate ventricular ejection with low oxygen demands by myocardium and high *P*_*md*_ allows adequate coronary perfusion. Therefore, the increased *P*_*ms*_/*P*_*md*_ ratio may cause a mismatch of the oxygen demand and supply in myocardium. In the present study, the rats after ECLS had higher *P*_*ms*_/*P*_*md*_ ratio than before (Table 2). Because the pulsatile nature of LV afterload could influence the LV function, we investigated the association of *P*_*ms*_/*P*_*md*_ with *τ*_*w*_/LVET and RI by using multiple linear regression analysis. We found that *P*_*ms*_/*P*_*md*_ was significantly affected by the magnitude of RI rather than the wave transmission time (*τ*_*w*_/LVET) (Fig. 3). Because |*P*_*f*_| and |*P*_*b*_| are the determinants of RI, the multiple linear regression analysis was performed to determine the influences of |*P*_*f*_| and |*P*_*b*_| on the *P*_*ms*_/*P*_*md*_ ratio. We identified a positive linear correlation between the *P*_*ms*_/*P*_*md*_ and |*P*_*f*_| (Fig. 4), indicating that the increased |*P*_*f*_| by ECLS impaired the matching condition of oxygen demand and supply in myocardium.

In the present study, the *PP* value was markedly larger after ECLS than before (Table 1). It has been considered that increased *PWV*, associated with vascular stiffening, and the consequent earlier return of reflected waves, prominently contribute to elevated *PP*^24,30,31^. However, we found that ECLS caused a decline in RM and RI, with no alteration in arterial *τ*_*w*_ and *τ*_*w*_/CL. Recent reports from Framingham investigators have demonstrated that increased |*P*_*f*_| is the predominant contributor to elevated *PP* with aging, with only modest contributions from wave reflections^26–28^. Thus, increased |*P*_*f*_| may be the predominant factor responsible for the elevated *PP* in rats after ECLS, with little alteration in wave transit time and a decline in wave reflection factor. The *P*_*ms*_/*P*_*md*_ ratio surged by ECLS was positively relevant to aortic *PP* (Fig. 5), indicating that the increased *PP* by ECLS impaired the matching condition of oxygen demand and supply in myocardium.

This study has some limitations. Because a rat’s blood volume is approximately 7% of that rat’s body weight^32^, the priming volume of our extracorporeal membrane oxygenator (ECMO) circuit was about 19–20 ml, which accounted for 61% of blood volume in the studied rats^22^. The priming volume used in this study was the minimum volume that we could attain in our model. As *Z*_*i*_ cannot be measured in conscious animals, it is difficult to evaluate the effects of anesthesia on the pulsatile hemodynamics in rats. The results reported here, therefore, pertain only to the measurements made in anesthetized rats. This condition might have induced changes in the aortic pressure profiles and introduced reflex effects not observed under ordinary conditions. The degree to which anesthesia influences the pulsatile hemodynamics in rats remains unclear. However, studies with other animal models suggest that the biological and experimental variability amid animals were small enough to neglect^33^. Moreover, the *Q*^tri^ was constructed by the measured aortic pressure wave to its assumed pairing flow signal. Although the approximated *Q*^tri^ may differ from the actual flow waveform, the use of this concept describing the arterial wave properties has been validated by Westerhof *et al*.^13^ and Chang *et al*.^17^.

## Conclusions

We demonstrated the mechanical defects of the vasculature in rats receiving ECLS based on the measured ascending aortic pressure and an assumed triangular flow. Although RI was diminished by ECLS, there existed a negative linear correlation between *P*_*ms*_/*P*_*md*_ and RI. In advance, we identified a positive linear correlation between *P*_*ms*_/*P*_*md*_ and |*P*_*f*_|, indicating that the increased |*P*_*f*_| by ECLS impaired the LV-arterial coupling. The *P*_*ms*_/*P*_*md*_ value was also positively associated with aortic *PP*, indicating that the increased *PP* by ECLS caused a mismatch of oxygen demand and supply in myocardium. All these findings suggest that |*P*_*f*_| was a predominant factor responsible for the impaired oxygen demand/supply ratio in the rats after ECLS. The advantage of this study is that an unknown *Q*^tri^ can obtain from the measured aortic pressure and the calibration of the flow is not necessary. Thus, it would provide a path to evaluate the arterial wave properties in the future clinical settings.

## Materials and Methods

### Animals and surgical procedure

The effects of ECLS on the pulsatile nature of the arterial system were evaluated in normal Wistar–Kyoto male rats weighing 450–550 g (NC + ECLS; *n* = 14). All rats were free access to Purina rat chow and water and housed two per cage in a 12-h light/dark cycle animal room. Periodic checks of the cages and body weights ensured that the food was appropriately administered. The experiment was conducted according to the *Guide for the Care and Use of Laboratory Animals*, and our study protocol was approved by the Animal Care and Use Committee of National Taiwan University.

The procedure for the implementation of ECMO in rats was conducted as described previously^22^. In brief, rats were anesthetized with 5% isoflurane/100% oxygen in an induction chamber (Serial number 4468, NorVap, Skipton, UK; Panion & BF Biotech Inc., Nangang Dist., Taipei, Taiwan), and then mechanically ventilated (tidal volume 7 mL kg^−1^, rate 40 breaths min^−1^, positive end-expiratory pressure 2 cmH_2_O; Model 131, New England Medical Instruments, Medway, MA, USA) by orotracheal intubation with 1.75%–2% isoflurane/0.7–0.8 L min^−1^ oxygen for maintenance. The rats were placed on a circulating warm water blanket (B401H, Firstek Scientific Co. Ltd., Xinzhuang Dist, New Taipei City, Taiwan; TP22G, Gaymar Industries, Inc., Orchard Park, NY, 14127 USA) with a heating lamp above for maintaining body temperature^22^.

The left femoral artery and vein were separately cannulated for arterial pressure monitoring (BIOPAC systems, Inc., Goleta, CA, USA) and drug administration^22^. The right femoral artery and the right external jugular vein were cannulated for the arterial inflow and venous outflow ports. Afterwards, heparin (500 UI) was administered to prevent blood clotting^22^. A high-fidelity pressure catheter (model SPC 320, size 2 French; Millar Instruments, Houston, TX, USA) was used to measure the pulsatile ascending aortic pressure from the isolated carotid artery of the right side. The electrocardiogram (ECG) of lead II was recorded using a Gould ECG/Biotech amplifier (Gould Electronics, Cleveland, OH, USA)^34^. Selective aortic pressure signals from 5 to 10 beats were averaged in the time domain, using the peak R wave of the ECG as a fiducial point^17^. Ascending aortic pressure signals were recorded in the anesthetized rats as a baseline before ECLS.

### Initiation and discontinuation of ECMO and intensive care unit phase

The ECMO device designed for rats consisted of an open venous reservoir (TERUMO$R$, Tokyo, Japan; 5-mL syringe), a membrane oxygenator (Micro-1 Rat Oxygenator; Dongguan Kewei Medical Instrument Co., Ltd, Guangdong, China), a heat exchanger (Radnoti Glass Technology Inc., Monrovia, CA, USA), silicone tubing (ID 1.6 mm), and a roller pump (Masterflex, Barrington, IL, USA)^22^. ECLS lasted for 30 min. The ECMO rate was initially quickly increased to the peak rate of 70 mL min^−1^—approximately the normal cardiac output in rats—and gradually decreased to approximately 20 mL min^−1^ to meet the venous outflow. In total, 12–15 mL complement fluid made from Plasma-Lyte A (Baxter, Deerfield, IL, USA) and 6% hydroxyethyl starch 130/0.4 (Voluven$R$; Fresenius Kabi, Bad Homburg, Germany) at the ratio of 1:1 was injected during ECLS to maintain the output of the circuit system.

The rats were weaned off the system after 30 min of ECLS, and the aortic pressure waves were measured at 30, 60, and 90 min after weaning (post-ECLS 30, 60, and 90 min). Blood transfusion or drugs such as bicarbonate, inotropes, or vasopressors did not perform on rats, and the rats were all humanely euthanized at the end of the experiment.

### Construction of the unknown flow wave by using a triangle

The unknown *Q*^tri^ was derived from the pressure waveform measured in the ascending aorta^13,14,17^. The start and end time points of LV ejection were identified as the intersection of two vertical lines near the foot of the pressure wave (the first vertical blue line in each of Fig. 1A–D) and near the incisura (the third vertical blue line in each of Fig. 1A–D), respectively^35^. The time at the peak of the triangle was derived from the fourth-order derivative of the aortic pressure wave^13,14,17^ (the pink curve in each of Fig. 1A–D). After ejection commenced, the first zero-crossing curve from above to below (the second vertical blue line in each of Fig. 1A–D) determined the peak of the triangle of blood flow, which was the inflection point of the pressure wave^13,14,36^. Thus, the uncalibrated *Q*^tri^ was approximated by a triangular shape (the green curves in each of Fig. 1A–D) and represented the corresponding flow wave of the aortic pressure signal.

### Impulse response function curve

*Z*_*i*_ was obtained from the ratio of the ascending aortic pressure harmonics to the corresponding harmonics from *Q*^tri^ by using a standard Fourier series expansion technique^11,14,35,37^. The aortic characteristic impedance (*Z*_*c*_) was calculated by averaging the high-frequency moduli of the *Z*_*i*_ data points from 4 to 10 harmonics. Arterial *τ*_*w*_ was computed using the impulse response function curve (the pink line in each of Fig. 1E–H)^38,39^, which was generated using an inverse Fourier transformation of the *Z*_*i*_ after multiplying the first 12 harmonics by a Dolph–Chebyshev weighting function with order 24^35,40^. One-half of the time difference between the appearance of the second reflected peak (long arrow) and the initial peak (short arrow) in the impulse response curve approximates the arterial*τ*_*w*_ in the lower body circulation^34,38,40,41^.

### Arterial wave separation analysis

The equations used to resolve the measured aortic pressure wave (*P*_*ao*_) into its *P*_*f*_ and *P*_*b*_ components were described as follows^35,42^:

1 $${P}_{f}(t)=\frac{{P}_{ao}(t)+{Z}_{c}\times {Q}^{{\rm{tri}}}(t)}{2}$$

2 $${P}_{b}(t)=\frac{{P}_{ao}(t)-{Z}_{c}\times {Q}^{{\rm{tri}}}(t)}{2}$$

The calculations of the *P*_*f*_ and *P*_*b*_ using *Q*^tri^(t) are depicted in Fig. 1I–L. The amplitudes (peak − trough) of the *P*_*b*_ and *P*_*f*_ are represented by |*P*_*b*_| and |*P*_*f*_|, respectively. The aortic reflection magnitude (RM) was then defined as the ratio of |*P*_*b*_| and |*P*_*f*_|: RM = |*P*_*b*_|/|*P*_*f*_|^13^, and the reflection index (RI) was calculated as RI = |*P*_*b*_|/(|*P*_*f*_| + |*P*_*b*_|).

### Statistical analysis

The results are expressed as median ± interquartile range (IQR). SigmaPlot 12.5 were used for statistical analysis. Wilcoxon signed-rank testing was performed to determine the statistical significance of the results between different time points for multiple comparisons of the effects of ECLS on arterial wave properties. Statistical significance was set as *P* < 0.05.
